# Osteopontin Is Required for the Early Onset of High Fat Diet-Induced Insulin Resistance in Mice

**DOI:** 10.1371/journal.pone.0013959

**Published:** 2010-11-12

**Authors:** Justin Chapman, Philip D. Miles, Jachelle M. Ofrecio, Jaap G. Neels, Joseph G. Yu, Jamie L. Resnik, Jason Wilkes, Saswata Talukdar, Divya Thapar, Kristen Johnson, Dorothy D. Sears

**Affiliations:** 1 Pfizer Inc., San Diego, California, United States of America; 2 Division of Endocrinology & Metabolism, Department of Medicine, University of California San Diego, La Jolla, California, United States of America; 3 Inserm U907, University of Nice-Sophia Antipolis, Nice, France; 4 Department of Reproductive Medicine, University of California San Diego, La Jolla, California, United States of America; University of Camerino, Italy

## Abstract

**Background:**

Insulin resistance is manifested in muscle, adipose tissue, and liver and is associated with adipose tissue inflammation. The cellular components and mechanisms that regulate the onset of diet-induced insulin resistance are not clearly defined.

**Methodology and Principal Findings:**

We initially observed osteopontin (OPN) mRNA over-expression in adipose tissue of obese, insulin resistant humans and rats which was normalized by thiazolidinedione (TZD) treatment in both species. OPN regulates inflammation and is implicated in pathogenic maladies resulting from chronic obesity. Thus, we tested the hypothesis that OPN is involved in the early development of insulin resistance using a 2–4 week high fat diet (HFD) model. OPN KO mice fed HFD for 2 weeks were completely protected from the severe skeletal muscle, liver and adipose tissue insulin resistance that developed in wild type (WT) controls, as determined by hyperinsulinemic euglycemic clamp and acute insulin-stimulation studies. Although two-week HFD did not alter body weight or plasma free fatty acids and cytokines in either strain, HFD-induced hyperleptinemia, increased adipose tissue inflammation (macrophages and cytokines), and adipocyte hypertrophy were significant in WT mice and blunted or absent in OPN KO mice. Adipose tissue OPN protein isoform expression was significantly altered in 2- and 4-week HFD-fed WT mice but total OPN protein was unchanged. OPN KO bone marrow stromal cells were more osteogenic and less adipogenic than WT cells *in vitro*. Interestingly, the two differentiation pathways were inversely affected by HFD in WT cells *in vitro*.

**Conclusions:**

The OPN KO phenotypes we report reflect protection from insulin resistance that is associated with changes in adipocyte biology and adipose tissue inflammatory status. OPN is a key component in the development of HFD-induced insulin resistance.

## Introduction

Insulin resistance associated with obesity, aging, and type 2 diabetes is an increasingly prevalent disease that affects skeletal muscle, liver, adipose tissue, and immune cells. In human and rodent models, obesity and insulin resistance are associated with macrophage infiltration and inflammation in the adipose tissue involving the secretion of inflammatory cytokines [1]–[3]. Adipose tissue inflammation impairs insulin signaling through negative feedback via cytokine-activated JNK, IKKβ and SOCS pathways [1]. Thiazolidinediones (TZDs) are used to treat insulin resistance in a variety of pathological states, including type 2 diabetes, polycystic ovary syndrome, and “syndrome X” [4], [5]. TZDs are ligands for peroxisome proliferator-activated receptor gamma (PPARγ), which is critical for maintaining proper metabolism in insulin target tissues [6]. PPARγ regulates the expression of many genes although the exact PPARγ target genes that modulate insulin sensitivity are as yet unidentified.

High fat diet (HFD) feeding is a common mode of inducing insulin resistance in rodents that rapidly causes progressive metabolic dysfunction [7], [8]. Insulin resistance in heart, adipose tissue, liver, and muscle, adipose tissue hypertrophy and inflammatory cell infiltration, and hyperinsulinemia are robustly observed as early as 1–3 weeks of HFD, with minimal to no total body weight gain [7], [9]–[13]. After prolonged HFD (16–20 weeks), these phenotypes are much more pronounced and accompanied by other severe metabolic dysregulation including dyslipidemia & ectopic triglyceride storage, hypo-adiponectinemia, adipose tissue hypoxia, cell death and remodeling, beta-cell decompensation, mild hyperglycemia, and deterioration of cardiac function [7], [8], [11]. The key molecules involved in the early stages of HFD-induced insulin resistance and adipose tissue inflammation and macrophage infiltration are not well characterized. Recent studies suggest an important role for osteopontin (OPN) in insulin resistance and macrophage recruitment to and regulation of inflammation in vascular and adipose tissue [14], [15].

OPN is a secreted, extracellular matrix-associated protein, with diverse biological activities many of which make it interesting for study in relation to insulin resistance and type 2 diabetes ([16], [17] and references within). For example, OPN is involved in cell migration and adhesion, macrophage activation, inflammation, tissue calcification, and matrix remodeling [18]. OPN is over-expressed in many pathophysiological states associated with insulin resistance and type 2 diabetes, such as, in the aorta of hyperglycemic diabetics, atherosclerotic lesions, activated macrophages, steatotic hepatitis, end-stage kidney failure, and osteoporosis. Positive regulators of OPN expression include cytokines, e.g., IL-6, IL-1β, INF-γ, TNFα, LPS, leptin, and angiotensin II, reactive oxygen species, and hypoxia [17]–[19], which dampen insulin sensitivity. PPARγ and/or LXR ligands have been shown to antagonize OPN expression in obese human adipose tissue [20], macrophage models [19], [21], mouse aorta [22], and fibroblasts over-expressing ectopic PPARγ together with an OPN promoter-driven reporter gene [15]. OPN is extensively and heterogeneously spliced, translated, phosphorylated, glycosylated, and proteolysed [23]–[25]. Post-translational modification and isoform expression of OPN varies by pathological state and cell type and differentially modulates its biological activity [23]–[28]. OPN binds to integrins and CD44 through which it can signal to downstream targets including phosphatidylinositol 3-kinase (PI3K), src kinase, and NFκB. OPN has been shown to be over-expressed in obese adipose tissue and adipose tissue macrophages from rodents and humans [20], [27] and is important for insulin resistance and adipose tissue macrophage infiltration after very long-term HFD (25 weeks) [15]. Given that OPN is a cytokine and regulator of cell migration and inflammation, OPN may play a role in the early development of insulin resistance.

In the current study, we measured OPN expression in adipose tissue from lean and obese rats and humans, before and after TZD treatment and found that OPN levels were increased with insulin resistance and normalized by TZD treatment. In order to investigate the role of OPN in the early onset of diet-induced insulin resistance, we compared wild type (WT) and OPN knockout (OPN KO) mice fed normal chow (NC) or HFD for 2–4 weeks. OPN KO mice fed NC had greater hepatic insulin sensitivity than WT mice and were protected from 2 week HFD-induced insulin resistance in skeletal muscle, liver, and adipose, as measured by hyperinsulinemic-euglycemic clamp and by insulin-stimulated Akt phosphorylation. Two week HFD increased plasma leptin levels, adipose tissue macrophages and cytokines, and adipocyte size in WT mice but these effects were absent or blunted in OPN KO mice. Short-term high fat feeding in WT mice was associated with altered OPN isoform expression but not change in total protein. Both HFD and OPN KO caused alterations on adipogenic and osteogenic potential of bone marrow stromal cells *in vitro*. Our studies demonstrate that OPN is required at an early stage of HFD-induced insulin resistance, before the onset of many pathophysiological features characteristic of extended high fat feeding.

## Materials and Methods

### Human sample studies

Archival samples of human adipose tissue RNA isolated during the course of a prior clinical study were used [20]. Subject-matched clinical data associated with the adipose tissue samples were compiled herein and correlated with OPN mRNA expression data. In the prior study, five lean, insulin sensitive and six obese, insulin resistant subjects were treated with pioglitazone (45mg/day) for three months. Clinical characteristics of the subjects are shown in Table 1. Before and after pioglitazone treatment, a subcutaneous adipose tissue biopsy was harvested from each subject and flash-frozen in liquid nitrogen, just prior to a 5 hr 60mU/m^2^/min hyperinsulinemic-euglycemic clamp study. Baseline plasma samples were drawn and hyperinsulinemic-euglycemic clamps were performed in the morning after a 10 hr fast as previously described [20]. The experimental protocol and sample studies were approved by the Institutional Review Board of the University of California, San Diego Human Research Protections Program. Informed written consent was obtained from each subject.

**Table 1:** Clinical Characteristics.

Zucker Rats	Lean, no Rx	Obese, no Rx	Obese, post-Rx
All male, 9 weeks of age, n = 7 per group			
Weight (g)	236±6	363±17*	387±14*
Fasting plasma glucose (mg/dL)	117.4±4.4	137.1±8.6*	124.8±4.0
Glucose disposal, Rd (mg/kg/min)	38.4±2.9	18.0±2.3*	30.6±2.9#
Fasting plasma insulin (pmol/L)	1.3±0.2	24.7±5.8*	4.8±1.4* #
Fasting plasma free fatty acids (mmol/L)	1.59±1.4	2.89±0.94	0.69±0.29* #
Fasting plasma triglyceride (mg/dL)	44.7±5.1	276.7±54.4*	108.0±16.1* #

Human Patients	Lean, pre-Rx	Lean, post-Rx	Obese, pre-Rx	Obese, post-Rx
Gender (male/female)	4/1		5/1	
Age (years)	45.2±8.7		53.8±2.1	
BMI (kg/m^2^)	23.1±1.0	23.6±1.2	36.3±2.3*	37.2±3.2*
Fasting plasma glucose (mg/dL)	90.4±3.6	87.6±3.1	97.7±4.5	91.7±2.5
Glucose disposal, Rd (mg/kg/min)	11.7±1.1	10.6±0.8	4.2±0.4*	5.9±0.5* #
Fasting plasma insulin (pmol/L)	10.3±1.4	10.1±2.2	22.1±3.9*	13.6±1.6#
Fasting plasma free fatty acids (mmol/L)	423±90	414±90	441±43	434±59
Fasting plasma triglyceride (mg/dL)	88.6±3.7	76.0±9.2	182.3±42.2	137.7±7.2*

Data are averages ± standard error. Rats were treated with pioglitazone for 3 weeks, humans were treated with pioglitazone for 3 months.

*p<0.05 vs. lean subjects.

# p<0.05 vs. pre-Rx subjects. Rx - pioglitazone treatment.

### Animal strains

Male C57Bl/6J WT mice (cat#000664) and OPN KO mice (B6.Cg-Spp1^tm2blh^/J, cat#004936) were purchased from Jackson Laboratories. The OPN KO mouse line has been backcrossed into the C57Bl/6J background >10 generations. Mouse diets were as follows: normal chow diet (12% kcal from fat; Purina 5001, LabDiet) and high fat diet (41% kcal from fat; TD96132, Harlan Teklad or, for SVC studies only, 60% kcal from fat; D12492, Research Diets). Mice were 4–6 months of age and age-matched in all studies. Archival tissue samples rat adipose tissue RNA samples from a larger study were used and group-matched clamp data associated with the adipose tissue RNA samples were compiled herein. In the original study, male lean Zucker (fa/+) and fatty Zucker (fa/fa) rats were used (Charles River). Lean rats were fed normal chow, fatty rats were fed normal chow or normal chow dosed to deliver 10 mg/kg/day pioglitazone for three weeks. All rats were nine weeks of age at time of terminal metabolic studies and tissue harvest. All animals were housed under controlled light (12∶12 light∶dark) and climate conditions. Animals had unlimited access to food and water. All procedures were performed in accordance with the *Guide for Care and Use of Laboratory Animals* of the National Institutes of Health and were approved by the University of California, San Diego, Animal Subjects Committee (protocol #s S02217, S00011, S99173).

### *In vivo* metabolic studies in rats

Insulin sensitivity was determined by hyperinsulinemic-euglycemic clamp, as previously published [29], [30]. A variable infusion of glucose (50% dextrose; Abbott Laboratories) was used, along with an infusion of tracer (0.16 µCi/min) and insulin (25 mU/kg^/^min, Novlin R; Novo Nordisk, Copenhagen). At the end of the clamp procedure, the animals were administered a lethal injection of sodium pentobarbital (100 mg/kg; Nembutal; Abbott Laboratories). Plasma glucose specific activity was measured after deproteinization with barium hydroxide and zinc sulfate [31]. Hepatic glucose output (HGO) and glucose disposal rate (GDR) were calculated for the basal period and the steady-state portion of the glucose clamp using the Steele equation for steady-state conditions [32]. Matched rat groups not subjected to hyperinsulinemic-euglycemic clamp studies were used for adipose tissue analyses. Tissues from these rats were excised after lethal injection, immediately flash-frozen in liquid nitrogen, and stored at −80°C for subsequent in vitro analyses.

### *In vivo* metabolic studies in mice

Insulin sensitivity was assessed using a submaximal hyperinsulinemic euglycemic glucose clamp technique and acute insulin stimulation as previously described [12]. During the clamp studies, glucose tracer was infused at 2 µCi/hr and insulin was infused at 3 mU/kg/min. The mice were conscious during the clamp and fully recovered after the procedure. Four days later, mice were fasted for 5 hr, anesthetized (isoflurane) to collect blood (cardiac puncture), and then euthanized (pentobarbital) to collect gastrocnemius muscle, liver, epididymal and subcutaneous adipose tissue. Portions of each tissue sample were flash-frozen in liquid nitrogen or fixed in Zn-formalin. Plasma glucose specific activity, GDR, and HGO were calculated as described above. Acute insulin stimulation was achieved by intraperitoneal injection of 6 hr-fasted mice (0.85 U/kg insulin). After 15 min., the mice were sacrificed and tissues were harvested as described above.

### Plasma and tissue analyses

Plasma insulin concentration was measured using the Insulin Ultrasensitivie (Mouse) EIA kit (Alpco, Inc.). Plasma FFA levels were measured enzymatically using a commercially available kit (NEFA C; Wako Chemicals USA). Triglycerides were measured using the Triglyceride-SL Assay (Diagnostic Chemicals Ltd.). Cholesterol was measured using the Chol kit and Roche/Hitachi analyzer (Roche). Other plasma and tissue lysates components were analyzed by SDS-PAGE-western blotting-chemiluminescence and ELISA. Total and serine-phosphorylated Akt were measured by western blotting (antibodies from Cell Signaling) and ELISA (Meso Scale Discovery). Plasma leptin, resistin, ACRP30, Mcp-1, and OPN (plasma and tissue) levels were determined by ELISA (R&D Systems). Western blotting was also used to measure tissue OPN (antibody developed by J.C. at Pfizer) and RBP4 (Adipogen, Inc.). Signal intensities of chemiluminescence autoradiographs were densitometrically quantified using a digital Kodak 3D Imagestation and associated digital image analysis software (Kodak,). IL-1β, IL-12p70, IFNγ, IL-6, IL-10, Cxcl1 and TNFα levels in plasma and tissue lysates were measured using a multiplex (7-plex) ELISA (Meso Scale Discovery).

### Fluorescence-activated cell sorting (FACS) of adipose tissue SVCs

Adipose tissue stromal vascular cells (SVCs) were isolated and analyzed by FACS as previously reported [11], [12] with minor modifications. Briefly, freshly harvested epididymal fat pads were separately rinsed and minced in DPBS +1% BSA then treated with 1mg/mL type II collagenase (Sigma) for 25 min in a 37°C shaking water bath. Adipose tissue cell suspensions were filtered through 100 µm mesh. SVCs were separated from floating adipocytes by centrifugation, incubated in RBC lysis buffer (eBioscience) for 5 min, then re-suspended in fresh DPBS +1% BSA. SVCs were incubated with Fc Block (BD Biosciences) for 15 min and then stained for 30 min with fluorescent-conjugated antibodies against F4/80 (Ab Serotec), CD11b (BD Biosciences), and CD11c (BD Biosciences). Cells were washed two times and re-suspended in DPBS +1% BSA and propidium iodide (Sigma). Presence of the fluorescent stains in the SVCs was analyzed using a FACS Calibur flow cytometer (BD Biosciences). Control SVCs preparations, including unstained cells, PI-only stained cells, and fluorescence-minus-one (FMO) stained cells, were used to set gatings and compensation.

### Adipocyte sizing

Excised fat pads were immediately fixed in Zn-formalin overnight, transferred to 70% ethanol, and subsequently paraffin-embedded. Paraffin sections stained with hematoxylin and eosin were used for determining cell size, as previously described [33]. All digital images of tissue sections were captured using the same microscope magnification. Microscopic fields with minimal non-adipocyte material were selected for quantitation of cell number per field. There was no apparent difference in non-adipocyte material in the sections between the mouse groups. Three fields were captured per mouse fat pad, from five mice in each group. Section images were visualized and cells per field image counted using ImageJ software (NIH freeware). Adipocyte size is represented by the inverse of the adipocyte number per field.

### Isolation and differentiation of plastic-adherent bone marrow stromal cells (BMSCs)

Mouse femurs were flushed with 1% FCS- containing DMEM low glucose medium. The washed bone marrow cells from the femurs were centrifuged for 10 min at 500×g and cultured for 14 days in Basal Mesenchymal Stem Cell (MSC) medium (Cambrex) supplemented with 1% glutamine (w/v), 100 U/ml Penicillin, 50 µg/ml Streptomycin, and 10% FCS. Differentiation of cultured BMSCs was conducted as previously described [34], [35] with slight modifications. For adipogenic differentiation, the BMSCs were plated in monolayer in MSC medium with the addition of 0.5µM dexamethasone, 50µM indomethacin and 0.5mM IBMX. The cells were grown for the days indicated and the media was replaced every three days. For osteogenic differentiation, BMSCs were plated in monolayer in αMEM medium containing 10% FCS, 0.5µM dexamethasone, 50µg/ml ascorbic acid, 10mM β-glycerophosphate and grown as above.

### RNA isolation and quantitation

Total RNA was isolated from human adipose tissue, mouse adipose tissue stromal vascular cells (SVCs) and mouse BMSCs using Trisol (Invitrogen), and from rat adipose tissue using the RNeasy Lipid Tissue Kit (Qiagen). *Human and rat adipose tissue RNA quantitation*: One step quantitative real-time PCR was carried out on 10 ng RNA. Primers and probes used were as follows: humanOPN: forward, 5′-AGTTTCGCAGACCTGACATCCAGT-3′; reverse, 5′-TTCATAACTGTCCTTCCCACGGCT-3′; probe, 5′FAM-TGGAAAGCGAGGAGTTGAATGGTGCA-TAMRA-3′; *ratOPN*: forward, 5′- TATCAAGGTCATCCCAGTTGCCCA-3′; reverse, 5′- ATCCAGCTGACTTGACTCATGGCT-3′; probe, 5′-FAM-TCTGATCAGGACAGCAACGGGAAGA-TAMRA-3′. Reactions were run on a 7900 Real-Time PCR System (Applied Biosystems) in a final volume of 20 µl containing 400 nM of the forward and reverse primers, 200 nM probe, 1× iScript Reverse Transcriptase and 1× iTaq RT-PCR Master Mix (BioRad). Reactions were performed in triplicate. Cycling parameters were as follows: 50°C for 10 min and 95°C for 5 min, followed by 40 cycles at 95°C for 10 sec and 60°C for 30 sec. Absolute quantitation was achieved by comparing to an OPN standard curve constructed using human or rat Universal Reference RNA standard (Stratagene). The standard curves had *r* ^2^ values of at least 0.99. GAPDH expression was used to confirm equal sample loading. *Mouse adipose tissue SVC and BMSC RNA quantitation*: RNA isolated from SVCs and BMSCs was converted into cDNA using reverse-transcriptase and dNTPs. For qPCR, 1 µL of a 25-fold dilution of the cDNA from specific reverse transcription reactions (above) was amplified using the LightCycler FastStart DNA MasterPlus SYBR Green I kit (Roche Diagnostics) with addition of 0.5 µM of each primer in the LightCycler 2.0 (Roche Diagnostics). Following amplification, a monocolor relative quantification of the target gene and reference GAPDH analysis was done to determine the normalized target gene/GAPDH mRNA copy ratios by the manufacturer's LightCycler Software (Version 4.0). The following primers were used: mouse GAPDH: forward 5′-CATCCCAGAGCTGAACG- 3′, reverse 5′-CTGGTCCTCAGTGTAGCC-3′; mouse OSX: forward 5′-CTCTCTTTGTCAAGAGTCTTAGC-3′, reverse 5′-AGAAAGATTAGATGGCAACGAGTTA-3′; mouse PPARγ: forward 5′-AGAGTCTGCTGATCTGCG-3′, reverse 5′-TCCCATCATTAAGGAATTCATGTCGTA-3′; mouse Akap2: forward 5′-AGACACAAGCATTCCCACTAT-3′, reverse 5′-CACCATCTCGGAGACCG-3′; mouse OPN: forward 5′- AGCCACAAGTTTCACAGCCACAAGG- 3′, reverse 5′- CTGAGAAATGAGCAGTTAGTATTCCTGC-3′. Primers for BMSC experiments were designed using the LightCycler Probe Design Software 2.0.

### Statistical analyses

Student's t test and ANOVA (with Tukey's post hoc test) were used for statistical analyses. P values for correlations were determined by linear correlation analysis (GraphPad Prism) using 2-tailed Pearson correlation coefficient. A p value cutoff of 0.05 was used to determine statistical significance.

## Results

### OPN is over-expressed and down-regulated by TZD treatment in human and rodent models of insulin resistance

**Figure 1:**
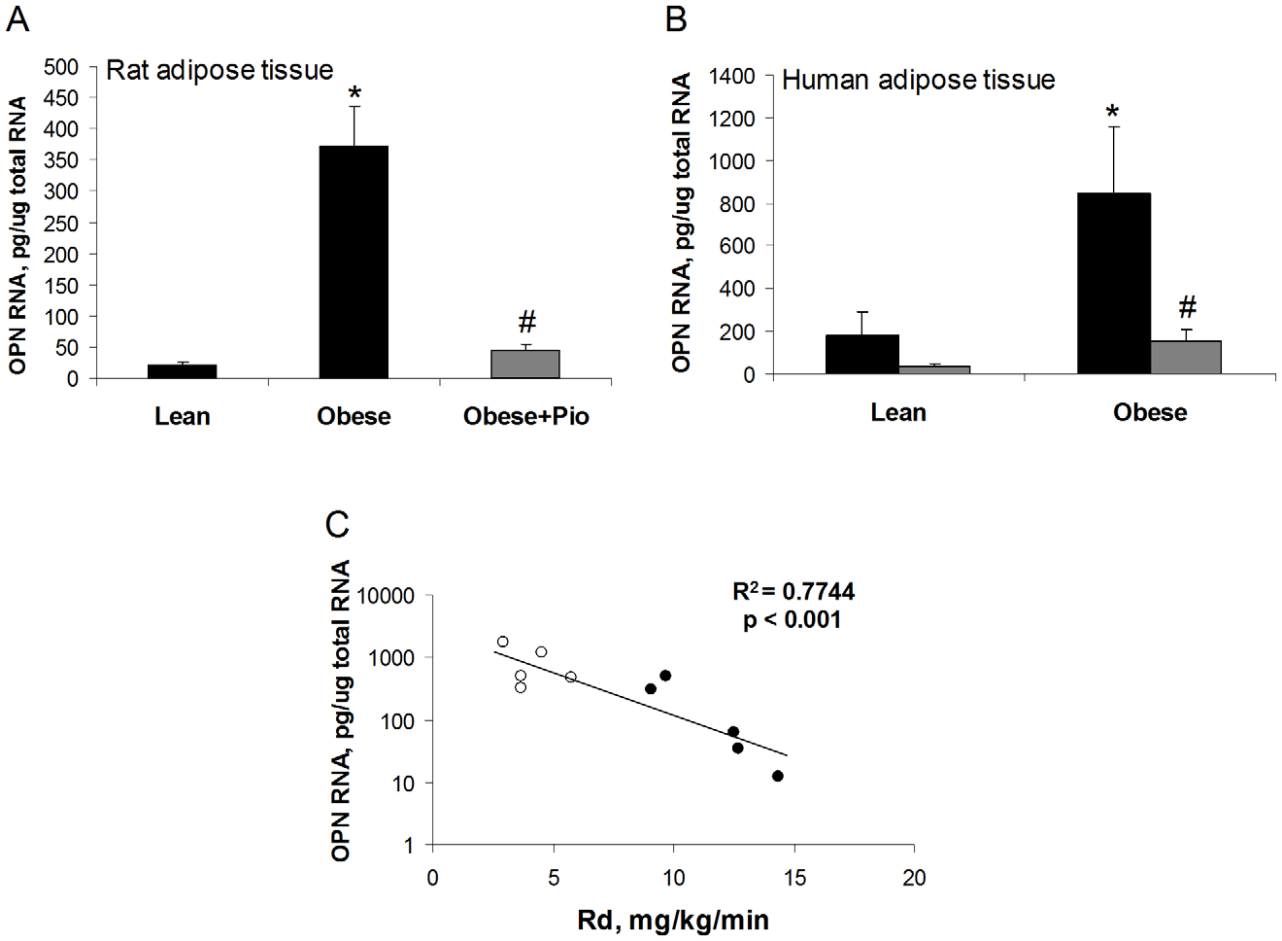
Adipose tissue OPN RNA levels are elevated in obese rats and humans and are suppressed by pioglitazone treatment. (A) Epididymal white adipose tissue OPN RNA levels from Zucker lean, obese, and pioglitazone-treated obese rats. 6 rats per group. (B) Subcutaneous white adipose tissue OPN RNA levels in lean subjects with normal insulin sensitivity and obese subjects with insulin resistance, before (black bars) and after (grey bars) pioglitazone treatment. 4–7 subjects per group. Values are averages ± standard error. * p<0.05 vs lean; # p<0.05 vs obese, before treatment. (C) Correlation between baseline adipose tissue OPN RNA levels and Rd in lean (filled symbols) and obese subjects (open symbols).

In the course of extensive microarray analyses of gene expression alterations associated with insulin resistance in adipose tissue from lean and obese humans and rats, we identified OPN as up-regulated in obesity and a TZD target gene [20], [30]. Herein, we used quantitative RT-PCR to compare OPN expression in adipose tissue from lean and obese humans and rats, before and after treatment with the TZD pioglitazone. Peripheral insulin sensitivity (rate of glucose disposal, Rd) of the rat and human groups was measured using the hyperinsulinemic euglycemic clamp method. Clinical characteristics of the rats and human subjects are detailed in Table 1. OPN expression in adipose tissue was elevated in obese, insulin resistant rats (17-fold) and humans (4.6-fold) compared to insulin sensitive lean controls (Figure 1A&B). Pioglitazone treatment improved insulin sensitivity in the obese rats and human subjects (Table 1) and normalized OPN expression in the adipose tissue from both species (Figure 1A&B). Notably, there was a significant correlation between adipose tissue OPN RNA levels and Rd in the combined, pre-treatment subject data (Figure 1C). Others report that adipose tissue OPN is over-expressed in obese humans and mice and in four genetic mouse models of obesity [15], [27], [36], [37]. Based on our own cross-species observations in obese human and rat models, we investigated the role of OPN in the development of insulin resistance.

### OPN KO mice are protected from HFD-induced insulin resistance

**Figure 2:**
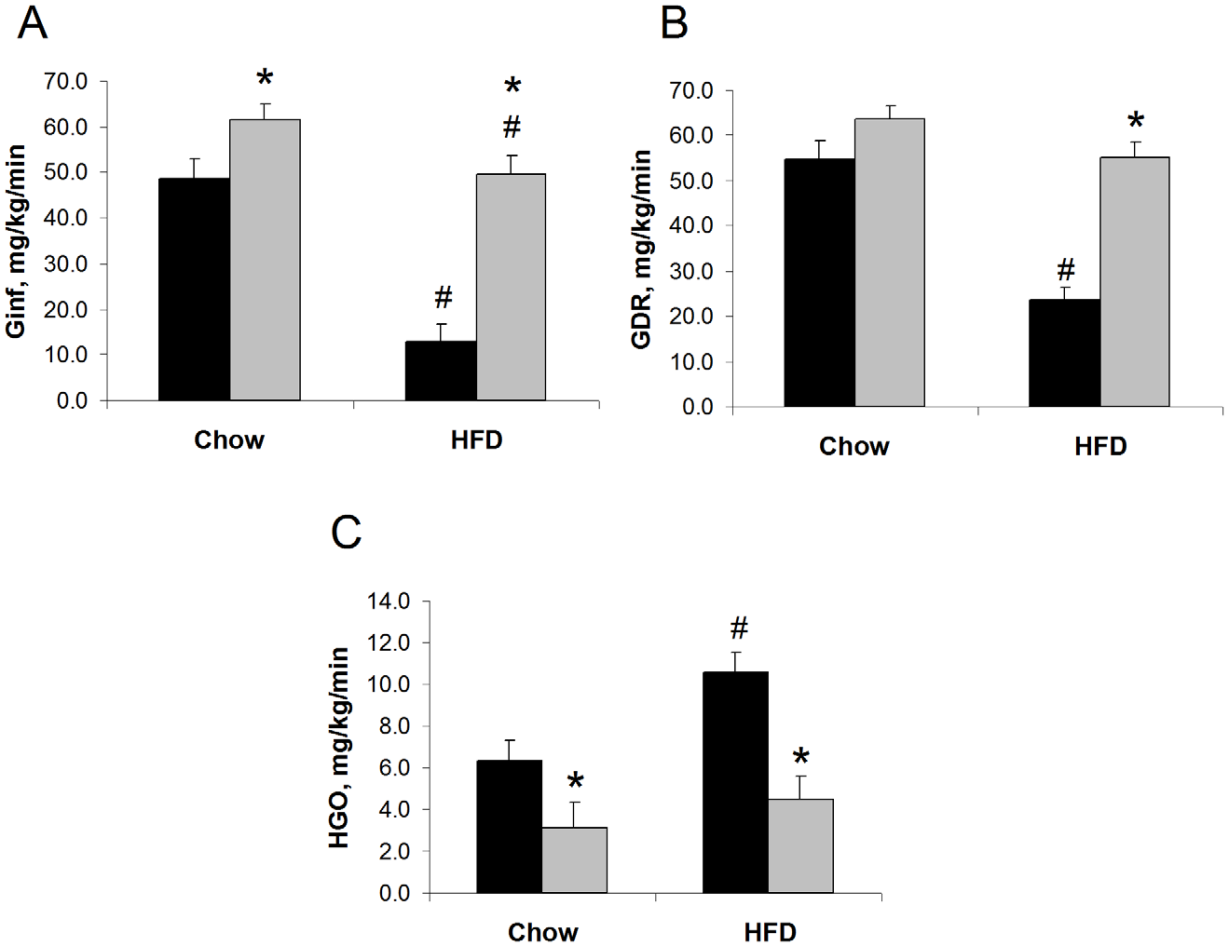
OPN KO mice are protected from HFD-induced insulin resistance. Euglycemic hyperinsulinemic clamp study results are shown. (A) Ginf, (B) GDR, and (C) HGO rates in WT (black bars) and OPN KO (grey bars) mice fed normal chow or HFD. Values are averages ± standard error. 7–9 mice per group. * p<0.05 vs diet-matched WT, # p<0.05 vs strain-matched, normal chow.

We studied the *in vivo* effects of whole-body OPN gene knockout in early onset, diet-induced insulin resistance using C57BL/6 WT and strain-matched OPN KO mice. We conducted euglycemic hyperinsulinemic clamp studies on WT and OPN KO mice fed normal chow (NC) or high fat diet (HFD) for two weeks. We observed significant differences in clamp data from the mouse strains fed either diet, reflecting greater insulin sensitivity in the OPN KO mice (Figure 2A–C). In the NC-fed groups, the average glucose infusion rate (Ginf) during the clamp was 27% greater in the OPN KO mice compared to WT mice and the average glucose disposal rate (GDR) during the clamp also tended to be greater in the OPN KO mice. Hepatic glucose output (HGO) during the clamp of NC-fed OPN KO mice was 52% lower compared to NC-fed WT mice. In the HFD-fed groups, OPN KO mice were protected from the severe HFD-induced decrease in Ginf (73%) and GDR (57%) and increase in HGO (66%) that we observed in WT mice. There was no significant difference in the basal glucose turnover rate (HGO = GDR) between WT and OPN KO mice (15.9±2.2 mg/kg/min and 16.2±2.5 mg/kg/min, respectively), nor between WT mice and HFD-fed WT mice (15.7±1.9 mg/kg/min). The clamp data indicate that the absence of OPN leads to improved hepatic insulin sensitivity when mice are fed NC and protection from hepatic and skeletal muscle insulin resistance when mice are fed HFD.

**Figure 3:**
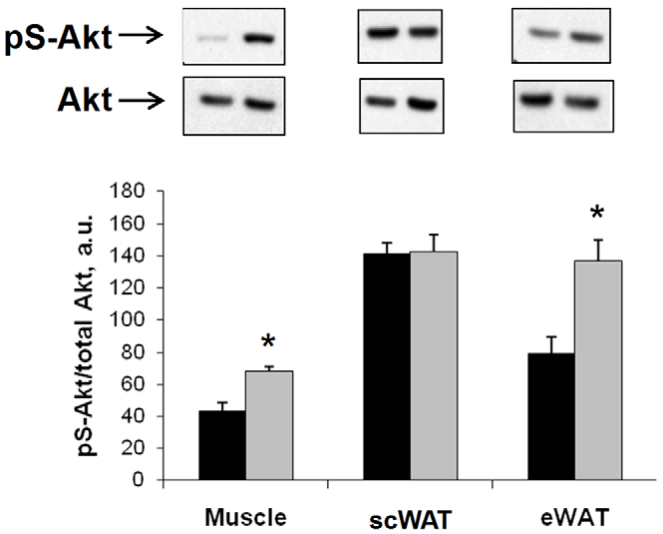
Insulin-stimulated Akt phosphorylation is enhanced in HFD-fed OPN KO mice. Phosphorylation of Ser^473^-Akt (pS-Akt) after 15 min *in vivo* insulin stimulation was measured in tissue lysates by ELISA and western blotting. Ratios of pS-Akt∶total Akt protein are normalized to basal levels in WT controls. WT fed HFD (black bars), OPN KO fed HFD (grey bars). A representative western blot from each tissue is shown directly above the matching bar graph. Muscle – gastrocnemius, scWAT – subcutaneous white adipose tissue, eWAT – epididymal white adipose tissue. Values are averages ± standard error. 8–10 mice per group. * p<0.05 vs WT.

In order to examine the effects of OPN KO on insulin signal transduction in HFD-fed mice, we conducted *in vivo* acute insulin-stimulation studies in the groups. Acute insulin-stimulated skeletal muscle and adipose tissue Akt phosphorylation results from HFD-fed mice are shown in Figure 3 and further demonstrate that HFD-fed OPN KO mice are more insulin sensitive that WT mice. Insulin-stimulated Akt phosphorylation in muscle was 58% greater in OPN KO mice compared to WT mice. Insulin-stimulated Akt phosphorylation was 73% greater in eWAT from OPN KO mice compared to WT mice but was not different between the strains in scWAT. These results are interesting in light of the fact that visceral adipose depots (e.g. eWAT) are much more susceptible to diet-induced insulin resistance than subcutaneous adipose depots.

Aside from differences in insulin sensitivity detected by euglycemic hyperinsulinemic clamp and acute insulin stimulation studies, we observed many similarities between HFD-induced changes in WT and OPN KO mice and observed that the two week HFD model of insulin resistance presents without many secondary abnormalities observed in longer HFD models. Total body weight was not different between the mouse strains and was unchanged by HFD (Table 2). Epidydimal white adipose tissue (eWAT) fat pad weights increased similarly in all animals on HFD, total weight and percentage of total body weight, and were not significantly different between the strains (Table 2). Fasting plasma insulin levels tended to be higher in the NC- and HFD-fed WT mice compared to the OPN KO mouse groups, but the differences were not significant by ANOVA (p = 0.068) (Table 2). Fasting plasma glucose levels were slightly elevated only in the HFD-fed OPN KO mice. We measured other plasma components in the four animal groups including OPN, adipokines, cytokines, chemokines, and lipids (Table 3). With the exception of total cholesterol, none of the plasma components in Table 3 were elevated in either the WT or OPN KO mice fed HFD. Total plasma cholesterol was elevated in both mouse strains fed HFD but was slightly lower in the OPN KO mice. Plasma OPN was not elevated after HFD in the mouse groups (Table 3).

**Table 2:** Mouse strain characteristics.

	WT NC	WT HFD	OPN KO NC	KO HFD
**Whole body weight, g**	27.5 (0.7)	29.3 (0.9)	27.2 (0.8)	28.7 (0.7)
**Epididymal fat pad weight, g**	0.24 (0.02)	0.71 (0.08)#	0.26 (0.02)	0.60 (0.05)#
**Epididymal fat pad mass, % body weight**	0.87 (0.08)	2.37 (0.25)#	0.95 (0.08)	2.13 (0.14)#
**Fasting plasma glucose, mg/dL**	162 (6)	172 (11)	177 (9)	203 (8)*
**Fasting plasma insulin, ng/mL**	1.65 (0.44)	1.89 (0.78)	0.62 (0.17)	0.61 (0.05)

Data are averages ± standard error. 7–10 mice per group.

*p<0.05 vs diet-matched WT,

# p<0.05 vs strain-matched NC.

**Table 3:** Tissue and plasma components.

	WT NC	WT HFD	KO NC	KO HFD
**Plasma Cytokines, etc.**				
**OPN (ng/mL)**	5108 (382.4)	5772 (568.5)	N.D.	N.D.
**ACRP30 (ug/mL)**	22.7 (1.7)	27.9 (4.0)	25.6 (3.2)	35.6 (6.3)
**MCP-1 (pg/mL)**	42.63 (1.1)	43.87 (14.2)	73.49 (12.8)	64.0 (9.6)
**IL-1β (pg/mL)**	43.1 (5.1)	22.4 (6.0)#	36.0 (2.8)	22.2 (2.0)
**IL-12p70 (pg/mL)**	59.5 (7.3)	39.4 (10.4)	50.6 (2.6)	37.8 (4.6)
**IFNγ (pg/mL)**	14.1 (1.7)	11.0 (3.4)	12.9 (0.8)	9.8 (1.3)
**IL-6 (pg/mL)**	218.5 (16.5)	179.7 (48.2)	187.1 (11.1)	167.0 (24.8)
**KC (CXCL-1) (pg/mL)**	84.4 (5.7)	67.2 (9.9)	93.5 (14.9)	63.5 (3.7)
**IL-10 (pg/mL)**	130.2 (1.1)	129.9 (1.6)	135.9 (1.9)	129.4 (1.1)
**TNFα (pg/mL)**	38.7 (3.5)	31.5 (4.1)	41.7 (3.2)	31.7 (1.2)
**Resistin (pg/mL)**	826.9 (66.5)	762.5 (92.7)	1067.0 (115.5)	788.8 (38.4)
**RBP4 (a.u.)**	2.3 (0.5)	2.1 (0.4)	1.9 (0.5)	2.0 (0.4)
**Tissue and plasma Lipids**				
**Total plasma cholesterol (mg/dL)**	78.4 (2.2)	147.8 (20.0)#	78.9 (2.5)	127.0 (3.6)* #
**Plasma triglycerides (mg/dL)**	31.0 (3.5)	28.8 (4.2)	39.2 (6.5)	24.6 (1.6)#
**Plasma free fatty acids (mmol/L)**	0.433 (0.025)	0.454 (0.099)	0.418 (0.024)	0.370 (0.027)
**Skeletal muscle triglycerides (mg/dL)**	15.3 (1.0)	15.7 (2.9)	12.5 (0.8)*	14.7 (0.9)
**Liver triglycerides (mg/dL)**	6.7 (1.1)	9.7 (1.3)	7.2 (1.0)	8.5 (1.2)

Data are averages ± standard error. 7–10 mice per group.

*p<0.05 vs diet-matched WT,

# p<0.05 vs strain-matched NC.

N.D. - not detected.

In several models of diet-induced insulin resistance, triglyceride accumulation is observed in muscle and liver. Table 3 shows that triglyceride levels were slightly, but significantly, lower in skeletal muscle from NC-fed OPN KO mice compared to WT mice. There was no difference in muscle triglyceride between the HFD-fed strains, neither was there a significant increase in triglyceride as a result of HFD. Liver triglyceride levels tended to increase in the HFD-fed WT and OPN KO mice, but this trend was not significant and was not different between the strains.

### HFD-induced adipocyte hypertrophy and hyperleptinemia are blunted in OPN KO mice

**Figure 4:**
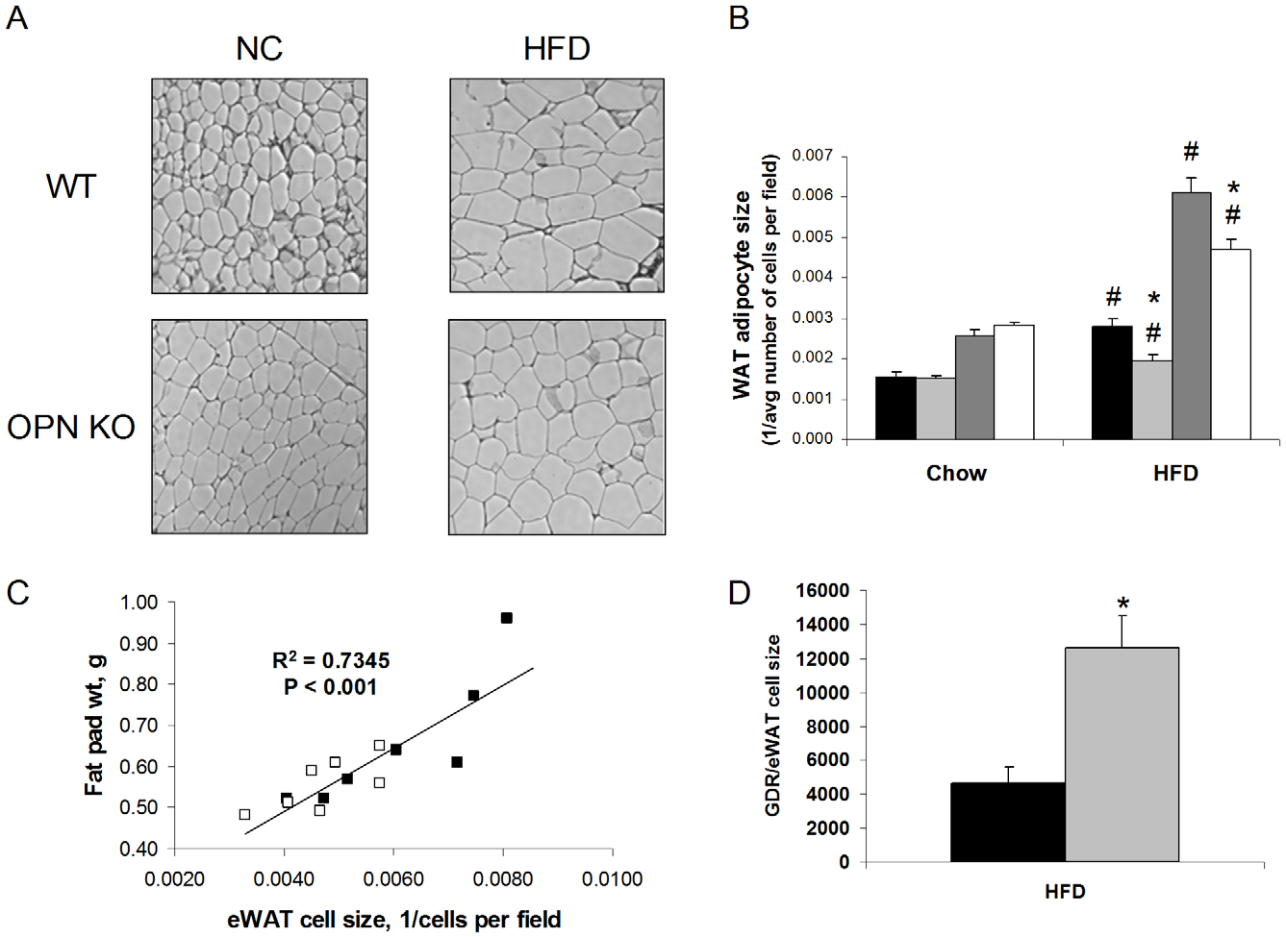
Adipocyte hypertrophy is blunted in OPN KO mice. (A) Representative histological images of eWAT from mouse groups. (B) Quantitation of subcutaneous WAT (scWAT) and epididymal WAT (eWAT) adipocyte size. WT scWAT (black bars), WT eWAT (dark grey bars), OPN KO scWAT (light grey bars), OPN KO eWAT (white bars). 7 mice per group. Values are averages ± standard error. * p<0.05 vs diet-matched WT, # p<0.05 vs strain-matched, normal chow. (C) Correlation of eWAT fat pad weight and adipocyte size in HFD-fed WT and OPN KO mice. WT (filled symbols), OPN KO (open symbols). 7 mice per group. (D) GDR normalized to eWAT cell size in HFD-fed WT (black bars) and OPN KO (grey bars) mice. 5–6 mice per group. Values are averages ± standard error. * p<0.05 vs WT.

We examined histological sections of the eWAT and subcutaneous WAT (scWAT). We observed that HFD-induced adipocyte hypertrophy in eWAT and scWAT from OPN KO mice was blunted 23% and 30%, respectively, compared to WT mice (Figure 4A&B). We did not observe any gross differences in extracellular matrix or other non-adipocyte cell structures when comparing the groups. Although eWAT fat pad mass was not significantly different between the HFD-fed strains (Table 2), eWAT mass and cell size were significantly correlated (Figure 4C). To demonstrate that the difference in insulin resistance between HFD-fed WT and OPN KO mice is not solely due to differences in their adipocyte cell size, we normalized the GDR values of the groups to their paired eWAT adipocyte cell size values (Figure 4D). Even after this normalization, there remains a dramatic difference in insulin sensitivity between HFD-fed WT and OPN KO mice.

**Figure 5:**
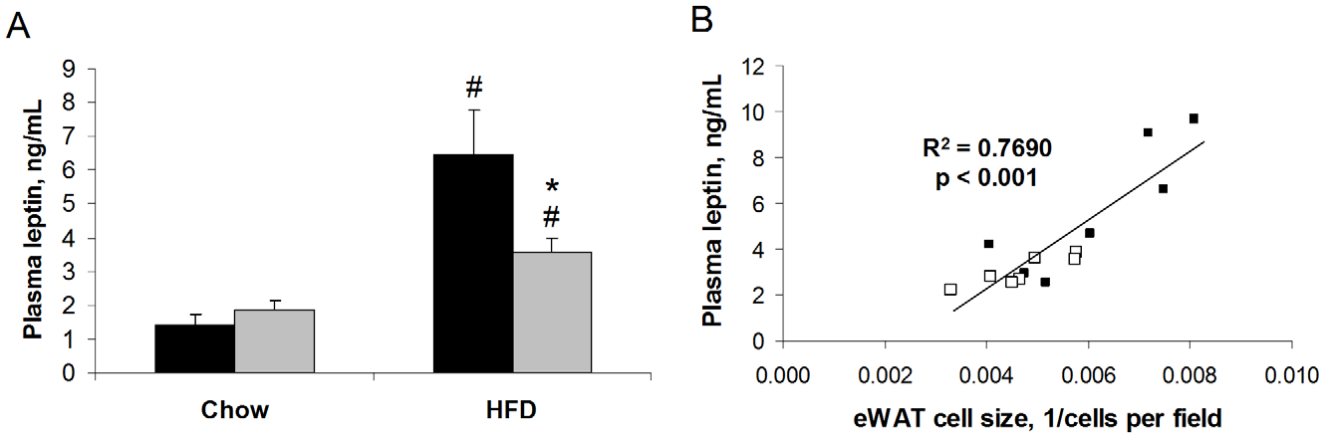
HFD-induced elevation of plasma leptin levels is blunted in OPN KO mice. (A) Leptin levels were measured by ELISA. WT (black bars) and OPN KO (grey bars) mice fed normal chow or HFD. Values are averages ± standard error. 8–10 mice per group. * p<0.05 vs diet-matched WT, # p<0.05 vs strain-matched, normal chow. (B) Correlation of plasma leptin levels with eWAT adipocyte size in WT and OPN KO mice fed HFD. WT (filled symbols), OPN KO (open symbols). 7 mice per group.

HFD caused increased plasma leptin levels in WT mice (4.6-fold) and that this was 45% blunted in OPN KO mice (Figure 5A). Again, although fat pad mass was not significantly different between the HFD-fed WT and OPN KO mice (Table 2), plasma leptin levels significantly correlated with eWAT adipocyte size (Figure 5B). This correlation supports our histological observation that adipocyte hypertrophy was blunted in the HFD-fed OPN KO mice, as plasma leptin levels have been shown to correlate with adipocyte size and fat mass in multiple species models of obesity [38]. We examined the eWAT and scWAT histological sections for adipose tissue macrophages using the macrophage-specific marker Mac-2. Mac-2 staining has previously been shown to increase in adipose tissue from obese mice and humans [39]. We did not detect any crown-like structures or differences in the number of Mac-2-stained cells when comparing sections from NC-fed and HFD-fed mice of either strain (data not shown). We further examined adipose tissue macrophage content using the more-comprehensive technique of fluorescence-activated cell sorting (FACS).

### HFD-induced adipose tissue macrophage infiltration and inflammation is absent in OPN KO mice

**Figure 6:**
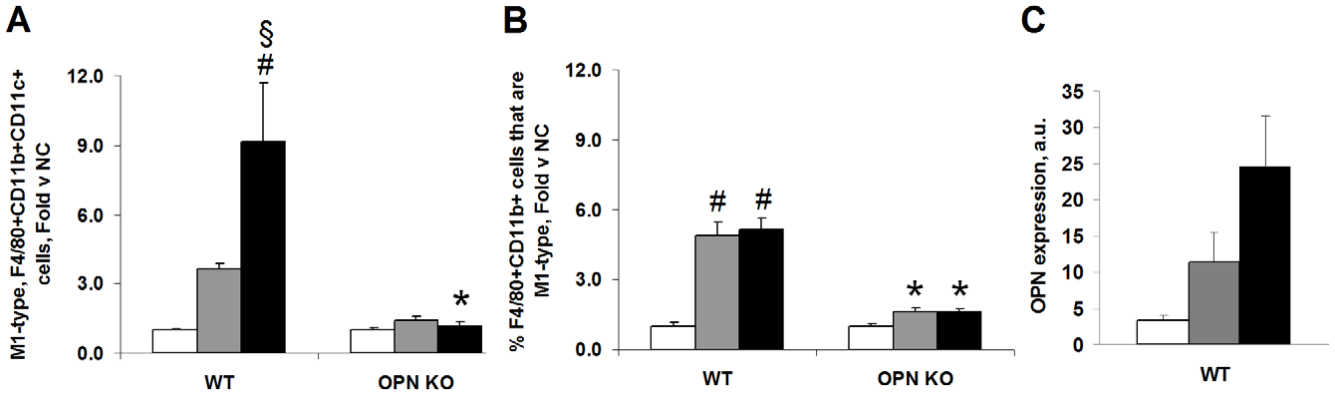
Inflammatory macrophage content and OPN expression in adipose tissue SVCs are blunted in HFD-fed OPN KO mice. (A) FACS detection of M1-type, F4/80+CD11b+CD11c+ pro-inflammatory macrophages in adipose tissue SVCs. Data are presented as fold change in the percentage of live adipose tissue SVCs expressing all three markers (F4/80, CD11b and CD11c) in the HFD vs normal chow (NC) groups. (B) FACS detection of percent of live adipose tissue SVC F4/80+CD11b+ macrophages that are CD11c+, M1-type. Data are presented as fold change vs strain-matched normal chow (NC) groups. (C) OPN expression increases in adipose tissue SVCs after HFD feeding in WT mice. SVCs were isolated during sample preparation for the FACS studies. NC (white bars), 2-week HFD (grey bars), 4-week HFD (black bars). 2–4 mice per group. *p<0.05 vs diet-matched WT, # p<0.05 vs strain-matched NC, § p<0.05 vs strain-matched 2-week HFD.

Adipose tissue is the site of localized macrophage infiltration and inflammation in obesity so, we examined macrophage content and cytokine protein levels in this tissue. We measured adipose tissue macrophage infiltration in eWAT-derived stromal vascular cells (SVCs) using FACS. The SVC fraction is heterogeneous cell population that includes immune-type cells, endothelial cells, and pre-adipocytes. FACS is a more sensitive and comprehensive method of detecting the distribution of specific cells types in adipose tissue compared to immuno-histochemical (IHC) methods. Cellular contents from the entire fat pad are used for FACS analyses in contrast to only micro-sections of whole tissue used for IHC analyses. In addition, co-expression of markers on single cells can easily be measured by FACS. Macrophages co-expressing the markers F4/80, CD11b and CD11c are increased in adipose tissue after HFD and these cells are pro-inflammatory, M1-type macrophages [11], [40]–[42]. We measured the percentage of live adipose tissue SVCs that express F4/80, CD11b and/or CD11c and the percentage that express all three markers, i.e., F4/80+CD11b+CD11c+ macrophages, in our mouse groups. The percentage of adipose tissue-derived F4/80+CD11b+CD11c+ SVCs was increased in HFD-fed WT mice fed HFD compared to NC-fed WT mice (Figure 6A). In contrast, adipose tissue-derived SVCs isolated from HFD-fed OPN KO mice exhibited no change in the percentage of SVCs expressing all three markers, F4/80+CD11b+CD11c+, compared to NC-fed OPN KO mice. Interestingly, we found that the percent of adipose tissue macrophages (F4/80+CD11b+ SVCs) that were M1-type (also CD11c+) increased significantly by 2 week and 4 week HFD (Figure 6B). This HFD-induced enrichment for M1-type cells in the total macrophage population was not observed in OPN KO adipose tissue. These data reveal that normal HFD-feeding-induced adipose tissue macrophage infiltration does not occur in OPN KO mice.

**Figure 7:**
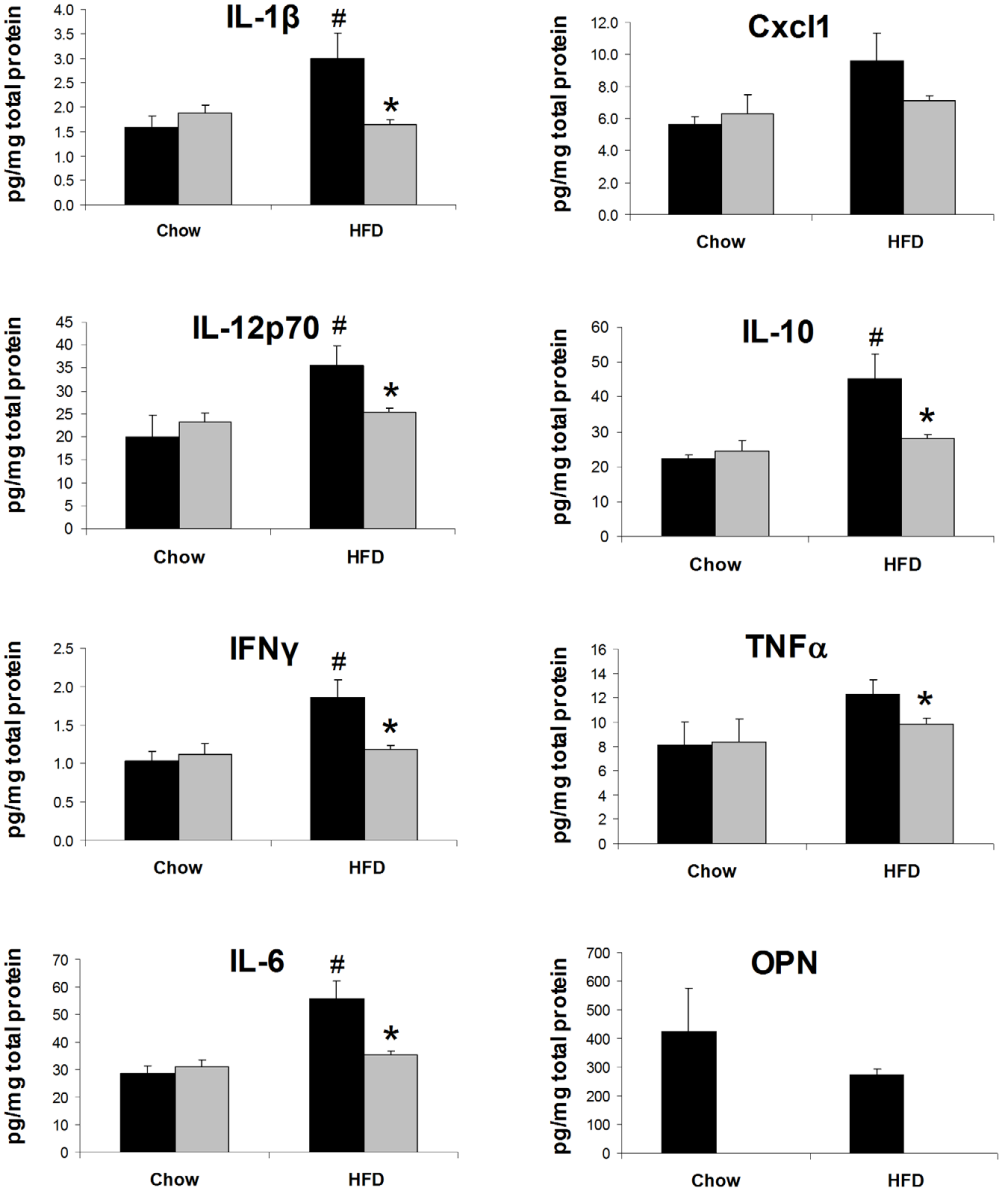
HFD-induced elevation of adipose tissue cytokine levels is blunted in OPN KO mice. Cytokine protein levels were measured in eWAT lysates from WT (black bars) and OPN KO (grey bars) mice fed normal chow or HFD. Values are averages ± standard error. 8–10 mice per group. * p<0.05 vs diet-matched WT, # p<0.05 vs strain-matched, normal chow.

We also measured cytokine protein levels in eWAT lysates from WT and OPN KO mice fed NC or HFD for two weeks. In eWAT lysates from WT mice fed HFD, we detected significantly elevated levels of IL-1β, IL-12p70, IFNγ, IL-6, and IL-10 (Figure 7). Cxcl1 (KC) and TNFα levels also tended to increase after HFD in WT mice but this increase did not reach statistical significance. HFD-fed OPN KO mice were completely protected from increases in eWAT lysate IL-1β, IL-12p70, IFNγ, IL-6, IL-10, Cxcl1 and TNFα levels. Together with our FACS data, it's clear that OPN KO mice are protected from HFD-induced adipose tissue macrophage infiltration and inflammation.

### HFD alters OPN isoform expression in adipose tissue

**Figure 8:**
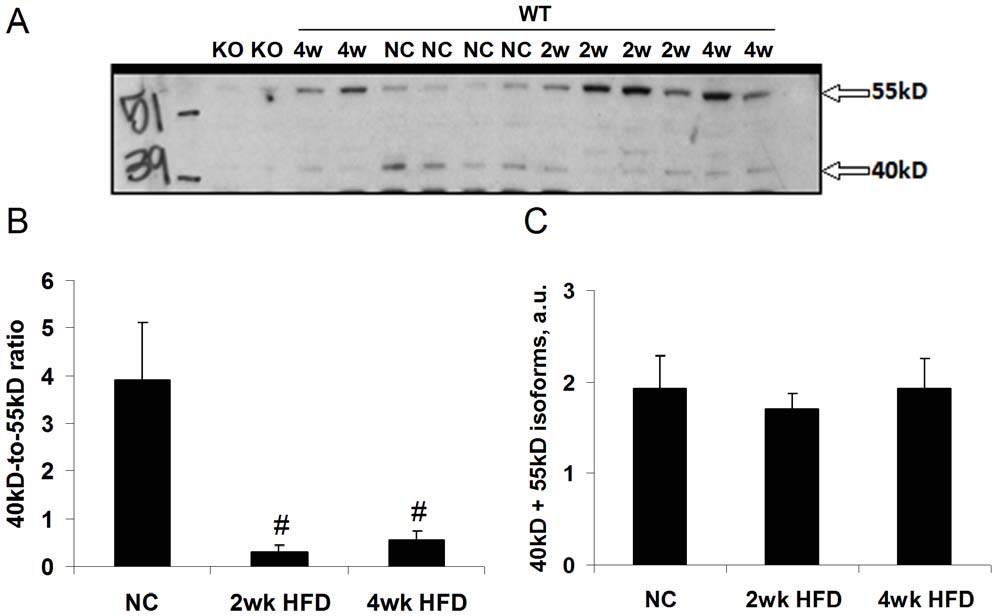
HFD alters adipose tissue OPN isoform expression. (A) Representative western blot of OPN isoforms with apparent molecular weights of approximately 40 and 55kD. (B) Paired ratios of 40kD∶55kD isoforms in all WT samples. (C) Sum of 40 and 55kD isoforms in all WT samples. Values are averages ± standard error. 4–10 mice per group. ^#^ p<0.05 vs normal chow (NC).

Although SVC expression of OPN mRNA was elevated after two- and four-week HFD in WT mice (Figure 6B), OPN protein levels, measured by ELISA, were unchanged in whole adipose tissue lysates from 2-week HFD-fed WT mice (Figure 7). In contrast, chronic models of HFD feeding and obesity have increased levels of adipose tissue OPN protein [15], [27], [37]. Because OPN in known to exhibit diverse alterations in isoform expression that are related to its biological activity, we evaluated potential OPN protein isoform expression changes induced by our short-term HFD protocol using SDS-PAGE/western blotting. HFD feeding induced significant alterations in OPN isoform expression (Figure 8A&B). NC-fed mice predominantly expressed a 40kD isoform and HFD-fed mice predominantly expressed a 55kD isoform. The sum of the two isoforms was not different between the groups (Figure 8C), in agreement with our ELISA data (Figure 7). Different OPN isoforms can be generated by heterogeneous splicing, translation, and post-translational modification which can vary by tissue and physiological condition and can alter its activity systems [23]–[28].

### OPN KO and HFD feeding alter bone marrow stromal cell osteogenic and adipogenic differentiation potential

**Figure 9:**
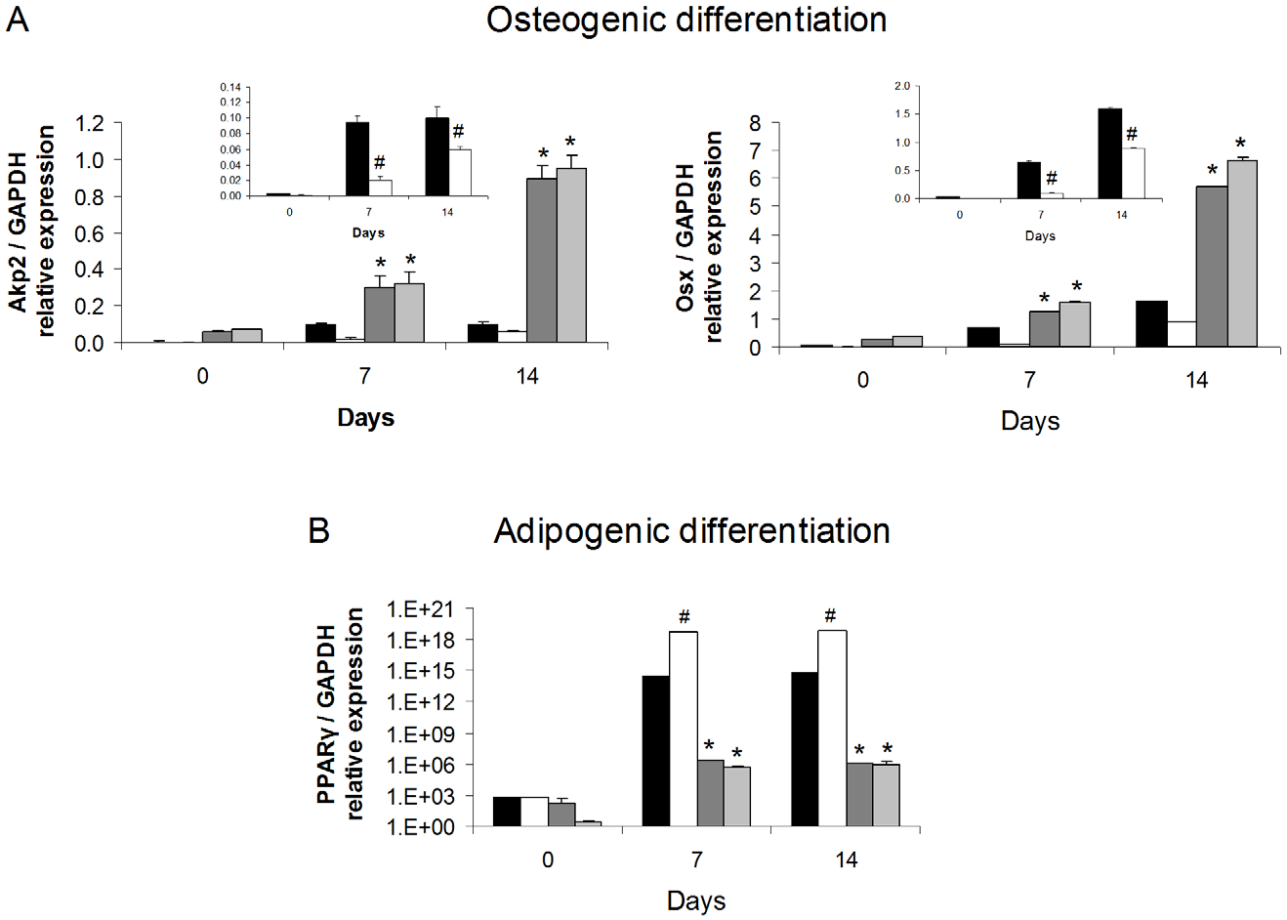
OPN KO and HFD effects on osteogenic and adipogenic differentiation of bone marrow stromal cells. BMSCs from the bone marrow of WT and OPN KO mice fed NC or HFD were cultured for 14 days and then subjected to osteogenic or adipogenic differentiation cocktails for the number of days shown. (A) Osteogenic differentiation was gauged by Akp2 and OSX RNA expression. Insert bar graphs are data from WT BMSCs shown separately. (B) Adipogenic differentiation was gauged by PPARγ RNA expression. Experiments were conducted in triplicate using BMSCs isolated from 4 mice per group. Gene expression data for all genes was normalized to GAPDH RNA expression. WT mice fed NC (black bars), WT mice fed HFD (white bars), OPN KO mice fed NC (dark grey bars), OPN KO mice fed HFD (light grey bars). Values are averages ± standard error. * p<0.05 vs diet-matched WT, # p<0.05 vs strain-matched, normal chow.

Bone marrow-derived mesenchymal stromal cells (BMSCs) are multi-potent and can differentiate along osteogenic, adipogenic and chondrogentic pathways [43]. OPN is a key regulator of bone biology and appears, based on our studies, to also be a key regulator of adipocyte biology. Therefore, we analyzed whether the absence of OPN expression and/or HFD would affect the propensity of BMSCs to differentiate through osteogenic and adipogenic pathways. We harvested BMSCs from the mouse groups and subjected them in parallel to either an osteogenic or adipogenic protocol. Progression through each differentiation program was measured by expression of marker osteoblast genes alkaline phosphatase (Akp2) and osterix (Osx) and the adipocyte gene PPARγ (Figure 9). Expression of the Akp2 and Osx during osteogenic differentiation was significantly greater in OPN KO BMSCs than in WT BMSCs (Figure 9A). HFD significantly blunted WT BMSC osteogenic differentiation but had no effect on OPN KO osteogenic differentiation (see inserts in Figure 9A). Expression of PPARγ during the adipogenic protocol was nine orders of magnitude greater in WT BMSCs compared to that in OPN KO BMSCs (Figure 9B). HFD significantly enhanced WT BMSC adipogenic differentiation (1000-fold more PPARγ expression) but had no effect on OPN KO adipogenic differentiation. It is striking that the two week HFD had such a profound effect on WT BMSC osteogenic and adipogenic differentiation potential after *ex vivo* culture for more than four weeks. It is possible that HFD exposure programs a differentiation potential bias in the BMSCs *in vivo*. Similar counter-effects of a pro-atherosclerosis diet on BMSC osteogenesis and adipogenesis have been observed [44]. Our data show that OPN deletion enhances osteogenic and inhibits adipogenic differentiation potential of BMSCs *in vitro* and, conversely, that HFD inhibits osteogenic and enhances adipogenic differentiation potential of BMSCs *in vitro*.

## Discussion

Insulin resistance is associated with chronic low grade inflammation in adipose tissue. Although the known biological roles of OPN include regulating inflammation, a role for OPN in regulating adipose tissue biology and/or whole body metabolism has recently been described [14], [15]. We found that OPN RNA expression was elevated in adipose tissue of obese insulin resistant rats and humans, was correlated with Rd in humans, and was normalized after TZD treatment. In global transcriptional profiling analyses, OPN is one of many inflammatory genes over-expressed in adipose tissue from humans [20] and five different mouse models of obesity [36]. OPN is expressed in many cell types [18]. OPN over-expression in obese adipose tissue is attributable primarily to macrophages and other stromal vascular cells [15], [27] but OPN is also expressed in adipocytes ([15], [45] and our data not shown). OPN expression is elevated in response to leptin, cytokines, and lipid modulators and is down-regulated by PPARγ and LXR ligands in human adipose tissue and other cell models ([15], [19]–[21], [46] and our data not shown). Our previous [20] and current findings suggest that normalization of OPN expression in adipose tissue may play a key role in TZD-mediated sensitization by reducing inflammation. Kiefer et al. have just published that an OPN-neutralizing antibody acutely administered *in vivo* inhibits diet-induced insulin resistance and inflammation [14].

We investigated the role of OPN in the early development of insulin resistance using a 2-4 week HFD feeding model [12]. Notably, the two week HFD was not associated with detectable changes in body weight, liver or skeletal muscle triglyceride, macrophage crown-like structures in adipose tissue, plasma FFA or triglyceride, or plasma inflammatory markers (with the exception of leptin). Our reported phenotypes of early onset high fat feeding (herein and [12]) are similar to those observed by Park, et al. in their timecourse study of HFD-induced insulin resistance [7]. Two-week HFD induced severe hepatic and skeletal muscle insulin resistance in WT mice, and hyperinsulinemia, hypercholesterolemia, and fat pad expansion in both mouse strains. OPN KO mice were protected from HFD-induced hepatic and skeletal muscle insulin resistance and had greater hepatic insulin sensitivity than WT mice when fed NC. OPN is over-expressed in nonalcoholic steatohepatitis (NASH) liver [47] and in liver from obese humans and mice, the level of which correlates with steatosis [37]. The OPN KO liver phenotype that we observe is supported by recent studies where acute OPN-neutralizing antibody treatment protected mice from diet-induced liver inflammation but not steatosis [14]. OPN KO mice also exhibited significantly enhanced insulin-stimulated skeletal muscle and eWAT Akt phosphorylation after HFD, compared to WT mice, which corresponds with their enhanced insulin sensitivity.

HFD increased plasma leptin levels in WT mice and this increase was blunted in OPN KO mice. We observed similar differences in the effect of HFD on leptin RNA expression in adipose tissue from these strains (data not shown). We also observed that the plasma leptin levels correlated with eWAT adipocyte size in both WT and OPN KO mice. In several obesity models, leptin levels are elevated, reflect adipose mass, and correlate with adipocyte size in humans [38], [48], [49]. TZDs and weight loss both reduce plasma leptin levels and adipocyte size [48], [50]. Leptin has inflammatory activity and can activate secretion of proinflammatory cytokines [38], [51]. Leptin induces Ser-318 phosphorylation of IRS1 in lymphocytes and skeletal muscle, inhibiting insulin signal transduction in muscle [52]. Cultured hepatocytes from *db/db* mice increase mRNA and protein expression of OPN after treatment with leptin and both the short-form leptin receptor and OPN were critical regulators in the *db/db* NASH model [53]. Thus, higher leptin levels in the plasma and, presumably, adipose tissue of HFD-fed WT mice may contribute to the multi-tissue insulin resistance we observe in WT vs. OPN KO mice. Other studies have shown that food and water intake, CO_2_ production, O_2_ consumption, physical activity, and thermogenesis were not different between the WT and KO strains fed either NC or HFD ([15] and D.T., D.D.S., A. Wynshaw-Boris, M. Street, unpublished observations).

Adipocytes from OPN KO mice were significantly less hypertrophic after HFD than the adipocytes from WT mice. Decreased HFD-induced hypertrophy of OPN KO adipocytes may be related to the decreased adipogenic potential of OPN KO BMSCs compared to WT BMSCs and is discussed in more detail below. In addition to secreting more leptin, larger adipocytes are less insulin sensitive, secrete more inflammatory cytokines and FFAs, and produce more reactive oxygen species and than smaller adipocytes [54]. We observe decreased insulin sensitivity and increased pro-inflammatory macrophage content and cytokine protein in eWAT from HFD-fed WT mice whereas these HFD-induced effects were absent in OPN KO mice. Increased cytokine secretion could be attributable to the increased pro-inflammatory macrophages, adipocytes, and/or other cell types in eWAT such as endothelial cells and preadipocytes. Leptin and OPN both induce inflammatory cytokine secretion [18], [38] and could be mediators of the increased cytokine secretion that we observe in the HFD-fed WT eWAT. Although total eWAT OPN protein did not change in HFD-fed WT mice, there was a significant HFD-induced shift in OPN isoform expression. OPN biological activity is modulated by differential splicing, translation, glycosylation, phosphorylation, and/or proteolysis that can give rise to various isoforms, as has been shown in other systems [23]–[28]. Thus, HFD-induced OPN isoform change may confer a biological activity to OPN that is important for initiating adipose tissue macrophage infiltration. Based on our human and rat expression data (within and [20]) and on the data of others [15], [27], [36], [37], we know that OPN expression does increase after chronic obesity and/or HFD.

Both *in vivo* and *in vitro* studies have demonstrated an inverse relationship between the differentiation of BMSCs into osteoblasts and adipocytes [43], [55], [56]. The balance between bone and adipocyte generation in bone marrow is affected by factors including aging, osteoporosis, and activation of PPARγ. In our studies, BMSCs isolated from OPN KO mice were significantly more osteogenic and less adipogenic than BMSCs isolated from WT mice which suggests that OPN is an important regulator of both differentiation pathways. A similar bone marrow phenotype was reported in mice expressing a deletion mutant FosB transgene [57]. Our data correlate with the reports that OPN KO mice have greater bone mineralization than WT mice [58] and are resistant to models of bone loss [59], [60]. Adipose tissue-derived stromal cells (ATSCs) and BMSCs have similar gene expression patterns and osteogenic and adipogenic differentiation potentials [61]. We speculate that the OPN KO ATSCs, like OPN KO BMSCs, may be less adipogenic than WT ATSCs. Little is known about the mechanisms regulating adipocyte hypertrophy. HFD-fed OPN KO mice have decreased adipocyte hypertrophy compared to WT mice, which may be due to decreased ability of the adipocytes to fully differentiate to maturity (reduced hypertrophic capability), and possible compensatory adipocyte hyperplasia to accommodate the high lipid load of HFD. Adipocyte hypertrophy and differentiation require extensive extracellular matrix remodeling [62]–[64]. Given the well-characterized role of OPN in extracellular matrix remodeling, OPN deficiency may impair adipocyte hypertrophy/differentiation through dysregulation of extracellular matrix. Notably, the effects of OPN deficiency on *in vitro* adipogenic BMSC differentiation suggest that the role of OPN in insulin resistance and adipocyte biology extends beyond it role in modulating immune cell function.

We have made the novel observation that two week high fat diet impedes subsequent *in vitro* BMSC osteogenic differentiation and enhances adipogenic differentiation in WT not in OPN KO mice. In the WT mice, this effect may be mediated by PPARγ ligands, either components or metabolites of the HFD, that have long term effects on the differentiation potential of BMSCs. This finding correlates with *in vivo* published studies relating a balance between bone marrow adiposity and bone density [43]. Interestingly, the high fat diet effect on BMSC differentiation potential was not observed in the OPN KO mice and further suggests that OPN is a positive regulator of adipogenesis and negative regulator of osteoblastic differentiation. The adipogenic and osteogenic differentiation potential of BMSCs from these mouse groups needs to be explored further using additional markers of differentiation.

In summary, we find that OPN is a key modulator of the early onset of high fat diet-induced insulin resistance in liver, muscle and adipose tissue. Our findings suggest that the mechanism by which the OPN KO mice are protected from HFD-induced insulin resistance involves reduced leptin expression, decreased hypertrophy of adipocytes, and suppression of inflammatory macrophage infiltration and cytokine secretion in adipose tissue. OPN deficiency also alleviates insulin resistance induced after chronic high fat feeding, in part, by reducing macrophage infiltration into adipose tissue [15] and acute neutralization of OPN inhibits obesity insulin resistance and inflammation [14]. OPN is novel participant in the early pathogenesis of diet-induced insulin resistance and a modulator of insulin target tissue biology. Our data and those from the references above strongly suggest that OPN may be an attractive therapeutic target for the treatment of human insulin resistance and type 2 diabetes.
